# Bio-semantic relation extraction with attention-based external knowledge reinforcement

**DOI:** 10.1186/s12859-020-3540-8

**Published:** 2020-05-24

**Authors:** Zhijing Li, Yuchen Lian, Xiaoyong Ma, Xiangrong Zhang, Chen Li

**Affiliations:** 1 School of Computer Science and Technology, Xi’an Jiaotong University, Xi’an, 710049 Shaanxi China; 2 Shaanxi Province Key Laboratory of Satellite and Terrestrial Network Tech. R&D, Xi’an Jiaotong University, Xi’an, 710049 Shaanxi China; 3 The Key Laboratory of Intelligent Perception and Image Understanding of Ministry of Education, Xidian University, 2 Taibai South Road, P.O. Box 224, Xi’an, China

**Keywords:** Bio-text-mining, Biological semantic relation, Knowledge base, Attention mechanism

## Abstract

**Background:**

Semantic resources such as knowledge bases contains high-quality-structured knowledge and therefore require significant effort from domain experts. Using the resources to reinforce the information retrieval from the unstructured text may further exploit the potentials of such unstructured text resources and their curated knowledge.

**Results:**

The paper proposes a novel method that uses a deep neural network model adopting the prior knowledge to improve performance in the automated extraction of biological semantic relations from the scientific literature. The model is based on a recurrent neural network combining the attention mechanism with the semantic resources, i.e., UniProt and BioModels. Our method is evaluated on the BioNLP and BioCreative corpus, a set of manually annotated biological text. The experiments demonstrate that the method outperforms the current state-of-the-art models, and the structured semantic information could improve the result of bio-text-mining.

**Conclusion:**

The experiment results show that our approach can effectively make use of the external prior knowledge information and improve the performance in the protein-protein interaction extraction task. The method should be able to be generalized for other types of data, although it is validated on biomedical texts.

## Background

Mol­ecular mechanism of biological semantic relations is fundamentally important in understanding biological processes and pharmaceutical design. Professional bio-reaction and pathway databases have taken much effort in the data curation and maintenance [1]; however, the manually curated have not been adequately exploited in text mining. We propose a novel approach to bring semantic information in the specialized knowledge bases (KBs) into the extraction of biological relations from the unstructured texts.

Biological relation extraction is the task of identifying the relationship between two or more entities from the biomedical literature. Previous research on biomedical relation extraction focused on protein-protein interactions (PPIs) [2]. PPI maps provide a valuable framework for a better understanding of the functional organization of the proteome [3]. Hua and Quan [4] extracted the PPI relation by using the shortest dependency path-based convolutional neural network (CNN) model. Their model makes use of the pre-trained word embedding for the PPI relation extraction task and could extract crucial features automatically. There are various tasks about biomedical relation extraction in the past. For example, the BioCreative III Workshop has several tasks that focus on text mining in biology, including two PPI tasks [5]. The goal of BioCreAtIvE - Critical Assessment of Information Extraction in Biology is to provide tasks focus on the prediction of protein interactions from biological articles [6].

The popularity of molecular biology study promotes the development of specialized KBs. Some KBs are manually managed by domain experts and therefore contain rich semantic information, such as molecular properties and their interactions. This information is stored and presented in a specific way (Such as BioGriD,^1^ Intact,^2^ UniProt,^3^ BioModels,^4^ DIP^5^). We assumed that the application of prior knowledge information could bring more useful features to the model. Thus, such enhancement would subsequently improve the performance of the model.

In this paper, we propose a novel system that extends a Bidirectional Long Short-Term Memory neural network (BiLSTM) by taking advantage of knowledge from KBs. The model acquires the information about the current word from KBs and utilizes the attention mechanism to determine the correlations between them. Choosing KBs that are more relevant to the experimental data is crucial to obtain better results. Functions vary among different KBs; in this paper, we want to choose two types of knowledge bases. One is about the entity itself. One is about substances that can react with the current object. The KBs we accept are UniProt and BioModels KBs, which provide domain-specific knowledge of entity attributes and relation compositions. UniProt KB is a public database, which offers rich semantic and functional information of proteins. It consists of two sections, including Swiss-Prot and TrEMBL. Specific protein functions, proteomic sequences, ontological relations, and other information can be retrieved from such a database. Data on biological functions are carefully annotated and reviewed concerning the original publications. BioModels Database is a free online database of biochemical reaction networks. The database includes signal transduction, metabolic reactions, and other information. In this paper, we propose a novel method for entity and relation extraction using KBs information and deep neural network. Here, we extract the semantic information of entities from the KBs and combine them with the entity representations trained from MEDLINE (The National Library of Medicine). Furthermore, additional semantic information about molecular reactions can be acquired from BioModels to improve the predictions of relation extraction.

We evaluate the approach on two information extraction tasks, i.e., entity and relation extraction. The experiment results show that our system is effective with regarding to the utilization of the information from KBs for both tasks on the BioNLP dataset and BioCreative dataset. The two evaluation datasets have different characteristics. The BioNLP SeeDev contains data on Arabidopsis. We select a subset of data and focus on the relations between genes and proteins. This paper assumes that the rich knowledge of the above and other similar databases can support not only biologists but also automated methods to improve information retrieval from unstructured texts. We believe that our approach is not limited to biological information; thus, it can be applied to other types of information in other fields as well.

### Related work

There are several computational methods to extract biomedical relations. Early identification is based on biological phenomena or markers. Gallet et al. [7] study the procedure identifies linear stretches of sequences by analyzing hydrophobicity distribution. With the popularity of statistical machine learning methods, they are used to predict PPI. Significant milestones using such a technique are discussed as follow. Yan et al. [8] apply the support vector machines (SVMs) for the prediction of PPI sites. Compared with traditional statistical machine learning methods, deep learning algorithms can process more complex data and automatically learn more useful features. Phan et al. [9] develop a method for extracting PPI networks between protein species, using a recurrent neural network (RNN) tailored for the alignment problem. Sun et al. [10] are the first to use a deep-learning algorithm for sequence-based PPI prediction, and the prediction performance is significantly improved.

Long Short-Term Memory (LSTM) network constitutes a type of time RNN, which is suitable for dealing with and predicting important events with relatively long intervals or delays in the time series. BiLSTM is a combination of two LSTMs, responsible for inputting forward and backward training sequences to the network. Li et al. [11] propose the “Bi-LSTM-RNN” model to extract biomedical entities as well as their relations simultaneously, and their results have been significantly improved. The neural networks have recently demonstrated this. It has proved to be very useful in tasks ranging from information extraction, question answering, and machine translations to other fields [12]. Therefore, in this work, we employ the BiLSTM network.

In recent years, the attention mechanism has been widely used in various tasks of NLP based on in-depth learning. “Attention is all you need,” published by Google’s machine translation team in 2017, uses a large number of self-attention mechanisms to learn text representation [13]. Lin et al. [14] propose a sentence-level attention-based model for relation extraction based on a multi-lingual cross-attention mechanism, which can sufficiently extract the relational patterns in different languages and enhance the learning of relational patterns. Verga et al. [15] propose a document level biological relation extraction model, which uses a transformer with self-attention submitted by Google to express input text. The results show that they have achieved advance results on BioCreative V Chemical Disease Relations. Zhou et al. [16] propose the Attention-Based Bidirectional Long Short-Term Memory Networks (AttBLSTM) for relation classification. In the SemEval-2010 relation classification task, this model is superior to most existing methods, using only word vectors. The mentioned examples demonstrate that attention mechanisms can help us solve problems and improve results to a certain extent. Therefore, in this paper, we also apply the attention mechanism.

More researchers have begun to make use of external KBs, and some of the approaches are using them in NLP tasks. Perera et al. [17] discuss the shortage of NLP techniques and demonstrate that the specific KBs can help to improve semantic annotation and information extraction. Yang and Mitchell [18] propose the KBLSTM by combining the KBs information from WordNet with BiLSTM. They employ the knowledge graph embedding approach to get the representations of the data from WordNet and NELL to improve the entity and event extraction on the ACE2005 corpus. Compared with Ratinov and Roth’s approach, this approach achieved better performance. However, due to the lack of flexibility in modeling context-specific knowledge, the model often makes incorrect predictions. Zhou et al. [19] leverage the prior knowledge with a memory network for relation extraction. They encode the triples in KBs into a continuous vector space by TransE. Zhou and Yang et al. [20] propose a neural network-based attention model for chemical-disease relation. In their work, they utilize both the context information in documents and KB information in KBs. Their overall system achieves comparable results with other state-of-the-art systems. Asada et al. [21] propose a neural method for drug-drug interactions. To improve the results, they also use the external drug molecular information; they increase the F-score by 2.39 percentage points.

In addition to the above methods, more and more researchers began to use the hybrid model to complete related tasks. They combine different techniques, learn the advantages of each method, and discard their disadvantages, to optimize the experimental results. The RNN and convolutional neural networks (CNN) are two popular and useful models for the relation extraction tasks. Each of them has its advantages and disadvantages. In that way, Peng et al. [22] propose the ensemble models, their system takes advantage of three models (SVM, CNN, RNN) and achieves the highest performance in the task. Zhang et al. [23] propose a hybrid model that combines the RNN and CNN to improve biomedical relation extraction. Their experimental results show that combining RNN and CNN can effectively improve biomedical relation extraction performance.

Our system integrates KBs and BiLSTM to extract biological entities and relations based on the mentioned methods. It is closely related to an attention-based BiLSTM introduced by Zhou et al. [16] However, they do not apply prior knowledge. It captures the most critical semantic information in the biomedical sentences. We extend the uses of attention-based BiLSTM by introducing the KBs information from the specialized biological databases.

## Results

We apply our proposed approach to the biomedical entity extraction and relation extraction tasks. In the course of the experiments, approximately 75% of entities in the corpus can be found in the UniProt KB; the BioModels KB can cover about 54% of objects. We try to search for more relevant KB information; however, there are still some entities whose information cannot be found in the two KBs. For such entities, we only use its word embedding, no extra information. Our performance matrices include precision (P), recall (R), and F1 values. The architecture is implemented by using PyTorch. For all of our models, we use a gradient descent optimization algorithm based on the AdaDelta (Zeiler [24]), with a learning rate of 1.0. The model parameters are regularized with a perminibatch L2 regularization strength of 10^− 5^. The BiLSTM state size is 200. We use a dropout rate of 0.3 in the BiLSTM layer. Also, we tuned the hyperparameters on the validation set by random search (Bergstra and Bengio [25]). Other parameters in our model are initialized randomly. In this paper, we use the BioNLP dataset and BioCreative dataset. For different data, there are some different hyper-parameters. For the BioNLP dataset, we set the minibatch size 10, for the BioCreative VI dataset, the minibatch size is 20. The amount of the two datasets is different. The amount of the BioNLP dataset is relatively small, so we set a small batch and a massive data amount corresponds to a large batch. Besides, the value of lr varies in a small range (0.8, 0.9, 1.0).

### Data

This study uses the data provided by the BioNLP-2016 competition and BioCreative VI. BioNLP is co-organized by the International Association of Computational Linguistics (ACL) and several internationally renowned universities (Cambridge University, Tokyo University, University of Manchester et al.). BioNLP has successfully held a series of competitions in biomedical tasks, such as the SeeDev task, which is set to study the event extraction of genetic and molecular mechanisms involved in plant seed development. Its entity and relation types are defined based on the knowledge model called Gene Regulation Network for Arabidopsis (GRNA)^6^. The task provides three data sets, the train data set, development data set, and the test data set. Our data is acquired from the SeeDev task, and we dedicate to work on the extraction of biological relations between proteins and genes. We only screen entities with gene and protein types, because only these two types of entities can be found in the UniProtKB. The details of the data are displayed in Table 1. We can see that there are two types of the entity, including gene and protein and eight types of relationships. Because the information in the KBs is only related to genes and proteins, so we choose only two types of entities. The BioCreative VI dataset aims to find protein-protein interactions that are affected by mutations (PPIm). It is as focused on humans as it is on precision medicine.

**Table 1:** The details of the data

Entity type	Gene
	Protein
**Relation type**	Is_Functionally_Equivalent_To
	Interacts_With
	Has_Sequence_Identical_To
	Transcribes_Or_Translates_To
	Regulates_Expression
	Is_Linked_To
	Binds_To
	Regulates_Molecule_Activity

### Entity extraction

We train the LibSVM and BiLSTM model to extract entities. The entity features for the SVM model include POS, Lemma, tree node depth, average word embedding. The BiLSTM model can also be trained with the CRF. CRF has been proved to be effective in extracting entities; we think the BiLSTM-CRF model is suitable for the entity extraction task. The features of the BiLSTM-CRF model are the same as those of the BiLSTM model. To better understand the validity of KB information, we train the model with and without KB information separately; the results are listed in the following tables. Due to the imbalance of data, it has a particular influence on the results of entity extraction.

From Table 2, we find that without any KB information, the two sets of results of the SVM and BiLSTM model are roughly the same. The features of the SVM model are plentiful, the abundant features probably lead to good results. There is no doubt that the CRF model is valid, compared with other work, BILSTM-CRF achieves good performance. To distinctly demonstrate our system, we add the information from UniProt and BioModels to make a comparison. To verify the effectiveness of our approach, we also add KB information to the BiLSTM-CRF method. From Table 3, with the information from KBs, all the models perform better than the previous work. We can see that the results of entity extraction have increased by 4.06% in BiLSTM with the UniProtKB and BioModels data compared to the one without any external information. In the meanwhile, the BiLSTM-CRF with KB information can raise the performance by 2.91% compared to the original implementation. The experimental results show that it is practical to introduce relevant information reasonably.

**Table 2:** Results of entity extraction without KB information

Model	P	R	F1
SVM	0.5914	0.5754	0.5833
CRF	0.5911	0.5915	0.5913
BiLSTM	0.5896	0.5760	0.5827
BiLSTM-CRF	0.6231	0.5825	0.6021

**Table 3:** Results of entity extraction based on different model with KB information

Model	P	R	F1
BiLSTM-Uni-Bio	0.6314	0.6154	0.6233
BiLSTM-CRF-Uni-Bio	0.6354	0.6282	0.6318

### Relation extraction

Li, Rao, and Zhang [26] propose the Litway, a system adopting a hybrid approach to use the LibSVM classifier with a rule-based method for the relation extraction in the SeeDev task of BioNLP-ST 2016. As a result, they achieved the best score. Thus, we use their approach as a benchmark for our system. For the SVM classifier, the candidate entity pairs are constructed within each sentence and validated by a multiclass classifier. The features for the SVM classifier include entity features, entity pair features, and rule-based features.

In relation extraction, we train a BiLSTM network without any KB information. For benchmarking, the Litway system (SVM) is also used to perform the same task. Besides, the attention-based BiLSTM network proposed by Zhou et al. [16] is chosen as the baseline, which outperforms most of the existing methods. As we mentioned before, Zhang et al. [23] propose a hybrid model that combines the RNN and CNN to improve biomedical relation extraction. We also utilize their method to make the comparison. In the BiLSTM experiment, we conducted two experiments with different inputs. One is the whole sentence containing the entity pair; the other is the sentence between the entity pair. From the results shown in Table 4, we find that using the BiLSTM network (use the sentences between the entity pairs as input) without any KB information obtains a better performance compared to another BiLSTM model (use the whole sentence as input). The results achieved by our system are compared with the best system in the SeeDev task of BioNLP-ST 2016 and the related excellent work. The SVM classifier achieves relatively good results. It has many rule points in the corpus, but there are no rules for the BiLSTM model. The BiLSTM-attention and RNN-CNN models also have a strong performance.

**Table 4:** Results of relation extraction without KB information

Model	P	R	F1
SVM (Litway)	0.4564	0.4343	0.4451
BiLSTM-attention	0.5024	0.4495	0.4512
RNN-CNN	0.5133	0.4199	0.4619
BiLSTM (the whole sentences)	0.4335	0.3654	0.3966
BiLSTM	0.4828	0.3930	0.4333

To verify our system, we do the following experiments: a BiLSTM network (use the whole sentence as input) trained with KB information from UniProt and BioModels; a BiLSTM network (use the sentences between the entity pairs as input) trained with KB information from UniProt and BioModels. The results are reported in Table 5. The experiments gain good results. Nevertheless, the BiLSTM-Uni-Bio model exhibits the best performance among all the models. Our approach, BiLSTM with KBs, has performed with a better F1 score by 2.3% compared to the original implementation of the best system without KBs. Combined with the two tables, reasonable use of KB information can effectively help the model to boost the results of relationship extraction. However, the basic model is also essential. Without any KB information, the RNN-CNN can show a strong performance. If we can incorporate the external information into the RNN-CNN model reasonably, we assume that the results can be boosted to some extent.

**Table 5:** Results of relation extraction based on different model with KB information

Model	P	R	F1
BiLSTM (the whole sentences)-Uni-Bio	0.4533	0.4006	0.4254
BiLSTM-Uni-Bio	0.5172	0.4575	0.4681

To further verify our approach, we also validate our system on the data of the GE4 tasks of BioNLP 2016. It is to prove that our approach is not limited to a single task and data, and the task fits our system. The GE4 tasks are open tasks, and we also work on relationship extraction. We choose two types of relationships, ThemeOf and CauseOf. Our results are shown in Table 6. The best results of the competition are shown in Table 7 (He et al. [27]). The best system proposes a two-stage method to detect the trigger, which also uses many features. Although the best system uses complex methods and functions, our system (BiLSTM-Uni-Bio) outperforms it in terms of both relationships.

**Table 6:** Results of relation extraction (BiLSTM-Uni-Bio)

Relations	P	R	F1
ThemeOf	0.49	0.58	0.53
CauseOf	0.51	0.32	0.35

**Table 7:** Results of relation extraction (the best system)

Relations	P	R	F1
ThemeOf	0.50	0.51	0.51
CauseOf	0.55	0.22	0.32

We want to use more data sets to verify the effectiveness of the method. The corpus from the BioCreative VI Track 4 is adopted. This part includes two tasks: document triage task and relation extraction task, namely the extraction of PPI pairs affected by gene mutations from literature. We select the relationship extraction task. The corpus contains the sum of 2097 Pubmed abstracts, the training data includes 100 abstracts, and the others are used for test data. We compare our work with other related excellent works, the results are shown in Table 8. The rule-based approach proposed by Chen et al. [28] achieves the highest rank in the PPIm extraction task. The rule-based approach is useful, but it has limitations--it can only target specific data. Tran et al. [29] use the CNN system to extract semantic features and also achieve relatively good results. Zhou et al. [20] try to incorporate prior knowledge into different models for the PPIm (CNN-KB, BiLSTM-KB). The CNN-KB system adds the entity (entities in the entity pairs) embeddings learned from KBs to each context word embedding. The BiLSTM-KB system also adds the entity (entities in the entity pairs) embeddings learned from KBs to each context word embedding. However, they divide the context into two parts, for the forward section, they add one entity embedding to each word embedding, for the backward sequence, they add the other entity embedding to each word embedding. The final representation of the input sentence is also added KB embedding. Yang and Mitchell [18] propose the KBLSTM by combining the KBs information from WordNet with BiLSTM. They employ the knowledge graph embedding approach to get the representations of the data from WordNet and NELL to improve the entity and event extraction. We also use their method to do the comparative experiment. Besides, we even choose two ensemble models, one is the RNN-CNN model, which is proposed by Zhang et al. [23], and another one is the CHEMPROT system (SVM, CNN, RNN) that is introduced by Peng et al. [22]. All the results of the mentioned methods are presented in Table 8. Without any KB information, our model is not the best. After adding the external information, it is evident that our whole system outperforms all the systems mentioned above. It can be seen that our system can still perform relatively better than other methods on different data sources.

**Table 8:** Results of relation extraction (BioCreative data)

Model	P	R	F1
Rule-based	0.3890	0.3010	0.3394
CNN	0.3653	0.2561	0.3011
RNN-CNN	0.3711	0.3288	0.3486
CHEMPROT	0.3732	0.3280	0.3491
CNN-KB	0.3602	0.3337	0.3464
BiLSTM	0.3215	0.3381	0.3296
BiLSTM-KB	0.3875	0.3157	0.3479
KBLSTM	0.3716	0.3276	0.3482
BiLSTM-Uni-Bio	0.3671	0.3331	0.3493

## Discussion

In this paper, we introduce KB information into the system. Through the analysis of the results, we believe that the introduction of KB has a specific effect. For example, in the sentence, “Two other LEC class genes, LEC2 and FUSCA3 (FUS3), are thought to share similar or overlapping functions with LEC1”, there is a relationship between “FUS3” and “FUSCA3”. In this case, the textual information, such as word embeddings and syntactic parsing, without any KB information, is still not enough to support extracting the relationship. When we add the KB information from UniProt and BioModels, we can obtain the relationship successfully. Also, the retrieved results can help us better understand the functional relationship between biological entities. In the sentence “It was subsequently shown that both VP1 and EmBP1, an Em1a-bindiNG BZIP protein, specifically interact with GF14, a 14-3-3 protein which may provide a structural link between these transcription factors”, after the extraction, we obtain the results that the functional relationship between “EmBP1” and “GF14” is “Interacts_With.” EMBP1 is one of the most widely studied dimerization protein in plants, and GF14 protein can participate in a series of stress response processes in plants (Schultz et al. [30]; Lu et al. 1994 [31]). From the extraction results, we can see this functional relationship.

There are also some problems with our system. In the experiment, the KBs only cover a limited number of entities recognized. Thus, the lack of semantic information is supposed to be the main factor of the relatively low improvement. Sufficient semantic information can be able to enhance performance. Meanwhile, more KBs with semantically cross-linking can further enrich the features for automated systems. In this paper, we only utilize two kinds of KB information, one is for the entity, and another one is for its relationship. We hope that more different KBs and different types of data can be applied to enrich the external information. At the same time, how to deal with the external information and how to lead it into the system are also crucial. It’s not enough to explore the semantic information stored in KBs only in simple ways. More useful approaches should be proposed to represent external information better.

Besides, many factors can determine the final results. According to the error analysis, there are three main factors. First, as we mentioned before, the related KB information can affect the results. Second, if the input sentence is too long (more than two sentences), the system may lead to errors. Therefore, it is crucial to process input sentences. Besides, the basic model itself is significant. Our proposed model still misclassifies the relationship between the entity pairs. Both of the RNN-CNN and CHEMPROT are performing strongly. If the external information can be reasonably added to the great model, the results can be greatly improved. Different models need different ways to do it.

## Conclusion

In this paper, we propose a BiLSTM network with the integration of KB information for improving the entity and semantic relation extraction tasks. First, we choose the appropriate KB information. In this work, we utilize UniProt and BioModels KBs, which are relevant to the experimental data. In the KBs, much information can be searched; we select the relevant information reasonably from it according to the experimental data and tasks. The prior knowledge information is represented by the word embedding; different ways are used to incorporate the KB information into the BiLSTM-based model. We have utilized the attention mechanism to facilitate the selection of KB information. The experiment results show that our approach can effectively integrate external KB information to improve the validity of biological information extraction. But the model and application of KBs still need to be improved. We hope that there can be further exploration both in theory and in methodology to make full use of the existing biological and other specialized KBs.

## Methods

**Fig. 1:**
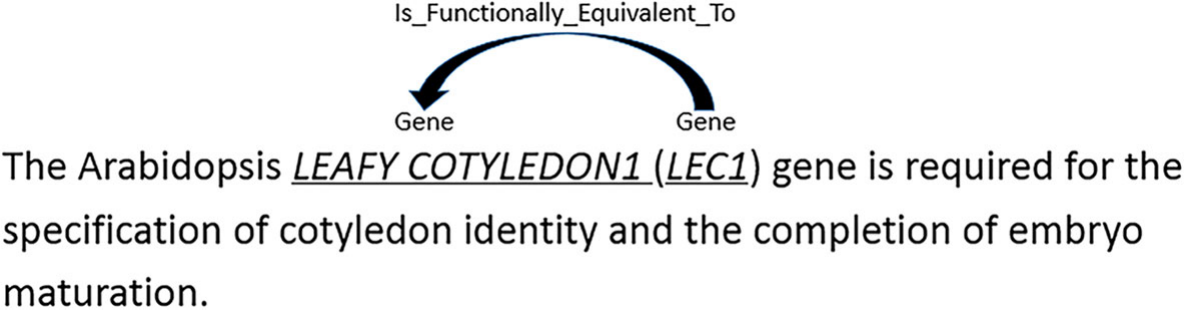
Relation extraction example in BioNLP-2016 competition. In this figure, we give an example of the relation extraction task in BioNLP-2016 competition

In this section, we introduce our approach based on the BiLSTM network with KB information integration. The example of identifying the entities and their relationship is shown in Fig. 1, where the sample sentence is “The Arabidopsis LEAFY COTYLEDON1 (LEC1) gene is required for the specification of cotyledon identity and the completion of embryo maturation.” In the example, “LEAFY COTYLEDON1” and “LEC1” are the same type, i.e., gene, and its relationship type is “Is_Functionally_Equivalent_To.” In the UniProtKB, the gene “LEC1” has alias as “NFYB9”, part of the description is “Lectin that may be involved in a cell recognition process.”. In the BioModels database, 77 entities can react to “LEC1”. So it is vital for our system to effectively determine which information from KBs is relevant (using attention mechanism).

**Fig. 2:**
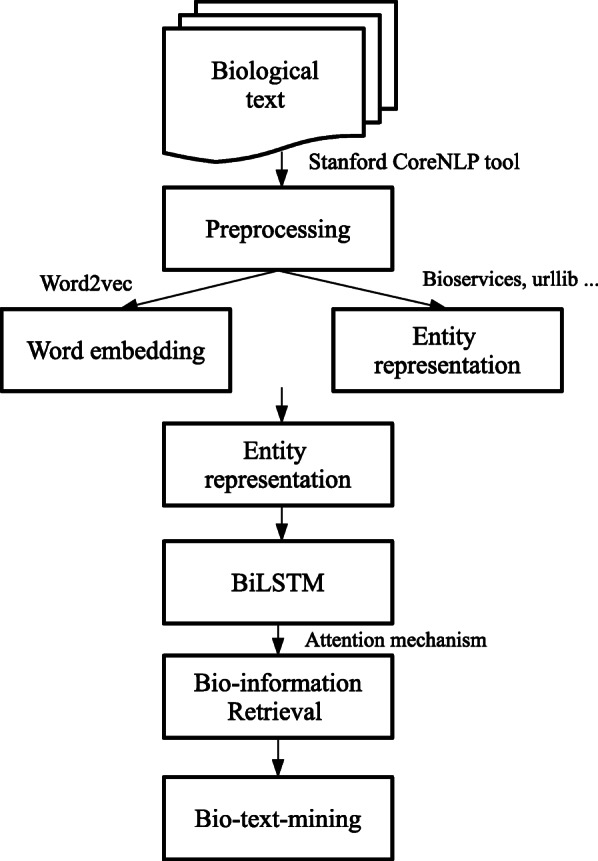
Flow chart of the proposed system. The processes of our system include preprocessing, word embedding, prior knowledge from UniProt KB, entity representation, BiLSTM, Bio-information retrieval (BioModels), and entity and relation extraction. For the prior knowledge from UniProt KB, we use Bioservices, urllib, BeautifulSoup tool does finish a series of processes. For the Bio-information retrieval (BioModels) part, we apply the attention mechanism to import the prior knowledge into the system. We use the method to do entity extraction and relation extraction. It is mainly about the relation extraction

The complete flowchart of our system is illustrated in Fig. 2. The first step after acquiring raw text is preprocessing. This step includes tokenization, sentence splitting, part-of-speech (POS), lemmatization, and parsing. The Stanford CoreNLP tool (Manning et al. [32]) is utilized in these operations. The result of preprocessing is to extract annotated entity pairs from each sentence. In the coming sections, we will introduce the entity representation, and bio-information retrieval, which are followed by the application of the system to entity extraction and relation extraction, respectively.

### Entity representation

**Fig. 3:**
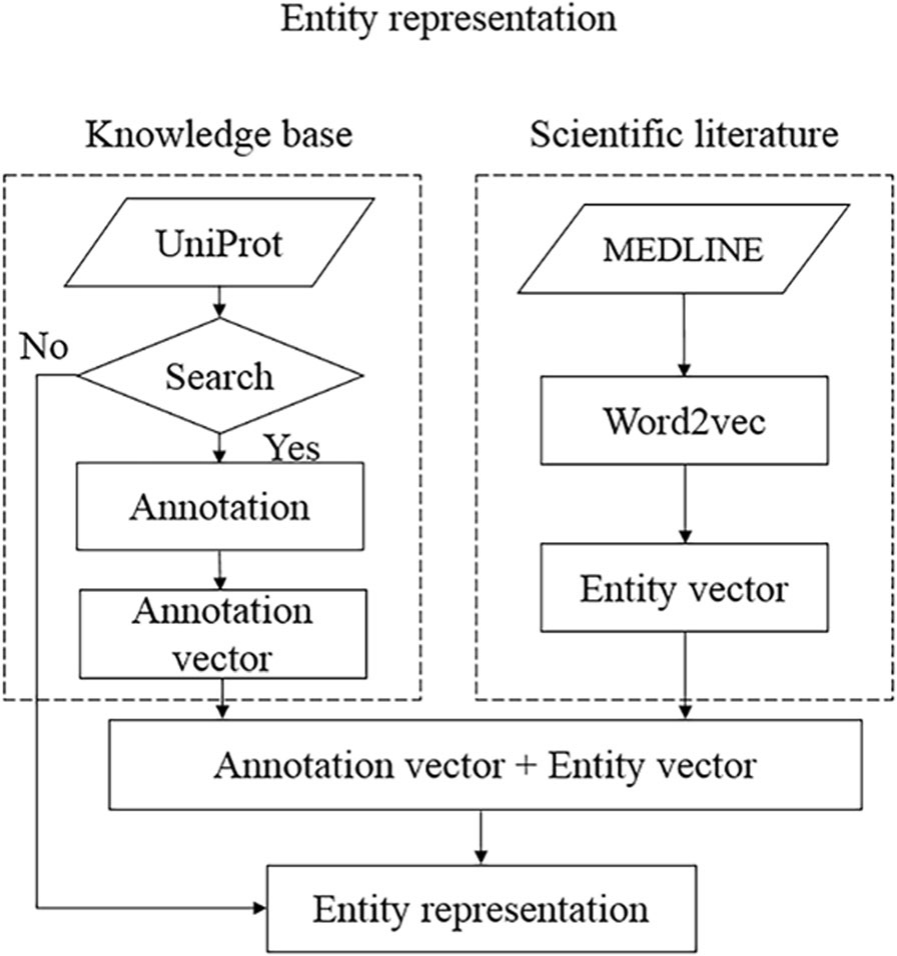
Entity representation. The entity representation includes information from both KB and scientific literature

The goal of entity representation is to represent each entity as a combination of extracted knowledge from UniProtKB and derived information from scientific literature specifically from MEDLINE, as illustrated in Fig. 3. For the scientific literature section, we first train the word embeddings, which include 1,701,632 vectors of distinct terms. They are represented by using word2vec (Mikolov et al. [33]) based on 10,876,004 MEDLINE abstracts. Thus, each word is described as a 200-dimensional vector. For the KB section, we obtain the information from the UniProtKB, which contains a great deal of information about the biological functions of proteins in the literature. Collecting embedding information from UniProt could be done by searching for UniProtID of the entity via the web service where we utilize bioservices version 1.5.2^7^--a Python package that provides access to many Bioinformatics web services, such as UniProt. It is a framework for conveniently implementing web services wrappers.

After that, the other two libraries, urllib^8^ and BeautifulSoup^9^ are used to access the corresponding web pages and obtain the annotations in the web pages, including functions, names and taxonomy information. An example of information we acquired from UniProt about entity “Contactin-2” is shown in Table 9.

**Table 9:** An example of obtaining the information of the entity “Contactin-2” from UniProt

Entity	Contactin-2
Function	In conjunction with another transmembrane protein, CNTNAP2, contributes to the organization of axonal domains at nodes of Ranvier by maintaining voltage-gated potassium channels at the juxtaparanodal region. May be involved in cell adhesion.
Recommended name	Contactin-2
Alternative name	Axonal glycoprotein TAG-1
	Axonin-1
	Transient axonal glycoprotein 1
	TAX-1
Gene names	CNTN2

For the same entity, we can query multiple corresponding IDs and numerous annotations. It is problematic to judge which annotations are more useful, so we combine all these annotations. After that, we match each word vector of the annotations from pre-trained word embeddings and add all these vectors. If we can not find the word from pre-trained word embeddings, we randomly assign vectors. In some cases, entities such as “LEC1” are both gene type and protein type. Therefore, we also obtain the type vector from the pre-trained word embeddings. Thus, multiple components are used to form the entity representation, including entity word vector, type vector, and annotation vector. Notice that the dimensions of these vectors are 200.

Through the previous steps, each word is represented as a 200-dimensional vector as the feature vector. Given a sentence *X* = (*x*_*1*_, *x*_*2*_, …, *x*_*T*_), the words are projected into a sequence of word vectors, denoted by (*e*_*1*_, *e*_*2*_, ..., *e*_*T*_) where T is the number of words.

### Bio-information retrieval

In this section, we utilize the attention mechanism to import information from BioModels into our system. The knowledge information at the time step t consists of the collection of entities associated with the current object collected from BioModels.

**Fig. 4:**
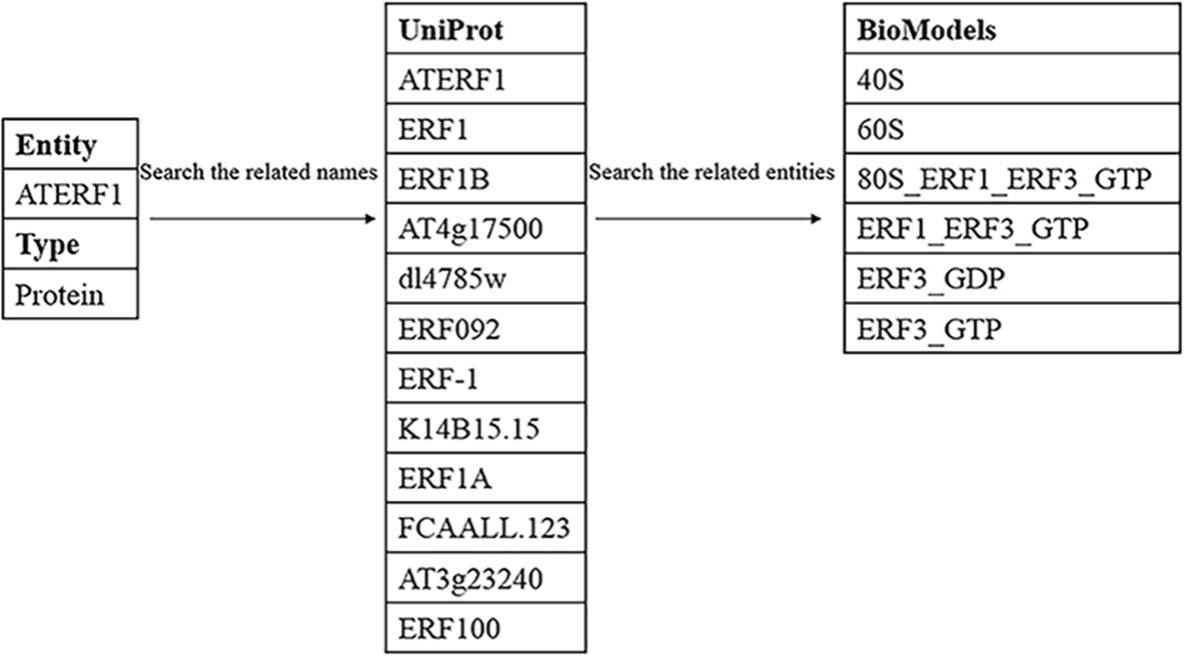
An example of the process of extracting the related entities of the entity “ATERF1” from BioModels. We give an example of a specific process of searching the related entities of the given entity

The process of obtaining bio-information includes the following steps. The BioModel database has 641 manually curated models and non-curated models, notice that we only consider the curated models which are stored as XML or SBML files. (1) Extract all the names of the given entities from UniProt (include protein names, gene names, short names). (2) Use each name we obtained to query the relevant objects from BioModels. The XML or SBML files contain much information for each model. We only use the “reaction” parts information under the “list of reactions” part. In this step, we extract the related entities only based on the “reaction” in each model to get a list of associated objects. We complete this process using The System Biology Markup Language (libSBML)^9^, a library for SBML files and data streams validation. Figure 4 illustrates a sample process of our system obtaining the relevant entities of the given entity “ATERF1” from BioModels. First, all the names of “ATERF1” are acquired by a search. In this case, there are 12 names of “ATERF1”. Then, we use all the names to search for the related entities of “ATERF1” from BioModels, and we can obtain six associated entities in this sample. Finally, we use the attention mechanism to choose the more significant entities for the system.

For each specific entity, only the relevant entities that exist in the reactions are considered and converted it into vectors for further processing.

**Fig. 5:**
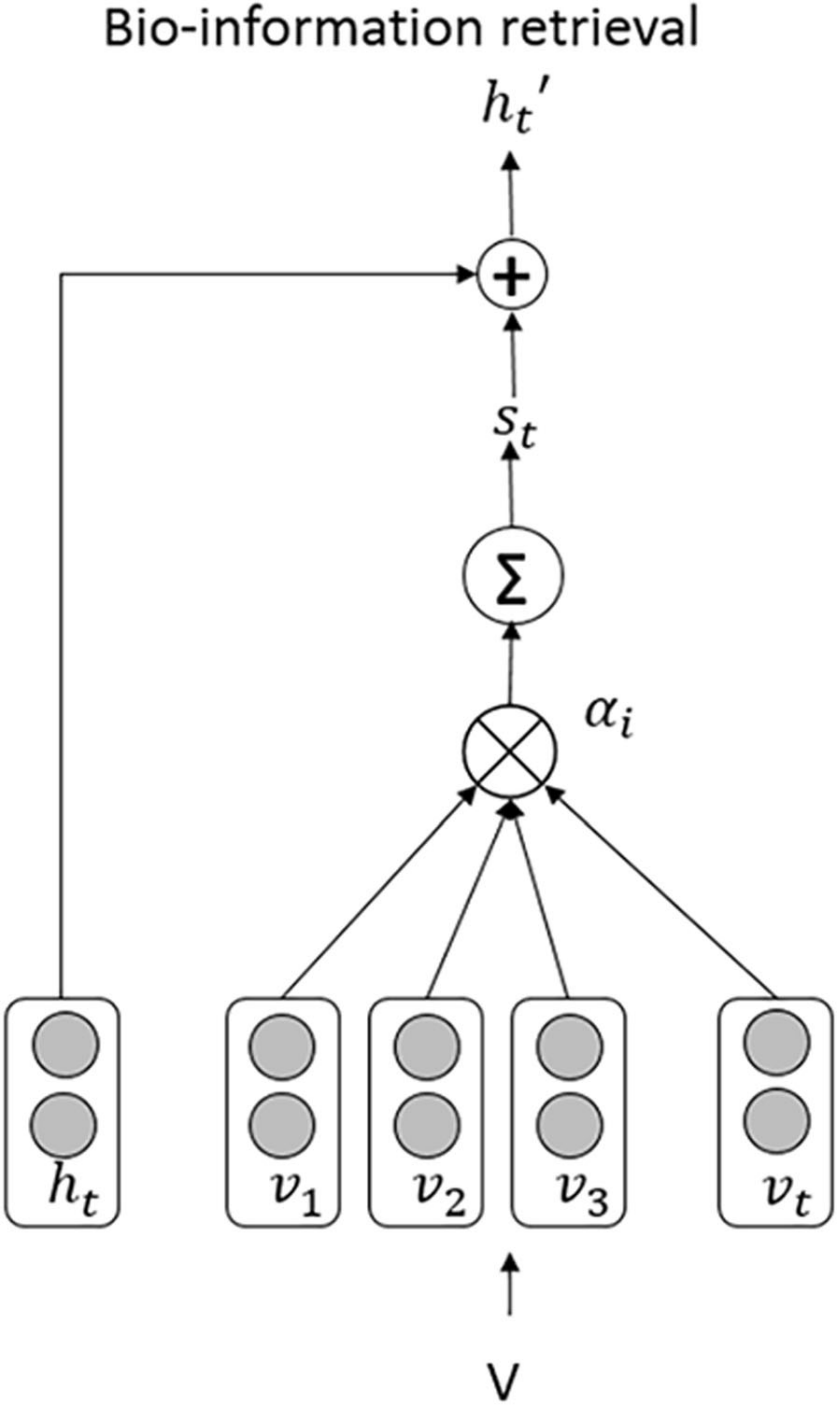
The architecture of the bio-information retrieval from BioModels. The attention mechanism of how to introduce the information from BioModels into the BiLSTM architecture

The collection of vectors, *V*, represents the bio-information retrieved from the KB. It consists of elements *v*_*i*_, which represent different associated entities, as shown in Fig. 5. The bilinear operator is used to compute the attention weight ***α***_***i***_ for each vector ***v***_***i***_; this reflects how the information from the KB is relevant to the current state vector ***h***_***t***_.

$$ {\alpha}_i\propto \exp \left({v}_i^T{U}_v{h}_t\right) $$

Uv is the parameter that has to be learned. According to Fig. 5, we can see that *s*_*t*_ is the state vector that integrates the KB information with the input at time t. *s*_*t*_ can be computed as:

$$ {s}_t=\sum \limits_{i\in V}{\alpha}_i{v}_i $$

We combine *s*_*t*_ and *h*_*t*_ to obtain ${h}_t^{\prime }$, which can be used to predict the entity type or the relationship between entity pairs. In the case where there are no associated entities from BioModels, V is an empty set; thus we set *s*_*t*_ = 0.

$$ {h}_t^{\prime }={h}_t+{s}_t $$

We use the softmax classifier to predict the label y^′^ from a set of labels Y from the entity or sentence. The state vector ${h}_t^{\prime }$ is used as input; therefore, y^′^ could be computed by:

$$ {p}_y= softmax\left(W{h}_t^{\prime}\right) $$

$$ {\mathrm{y}}^{\prime }=\arg \underset{y}{\max }{p}_y $$

### Entity extraction

To demonstrate that our system is not limited to a specific task, we apply our approach to extract the entities. In entity extraction, the entity could be composed of several words, so each word within an entity in the training set is tagged to represent the token’s position. There are four tags in our proposed approach, including “B,” “I,” “O,” and “E,” which state that the token is at the beginning, on the inside, on the outside, or at the end of the entity respectively.

**Fig. 6:**
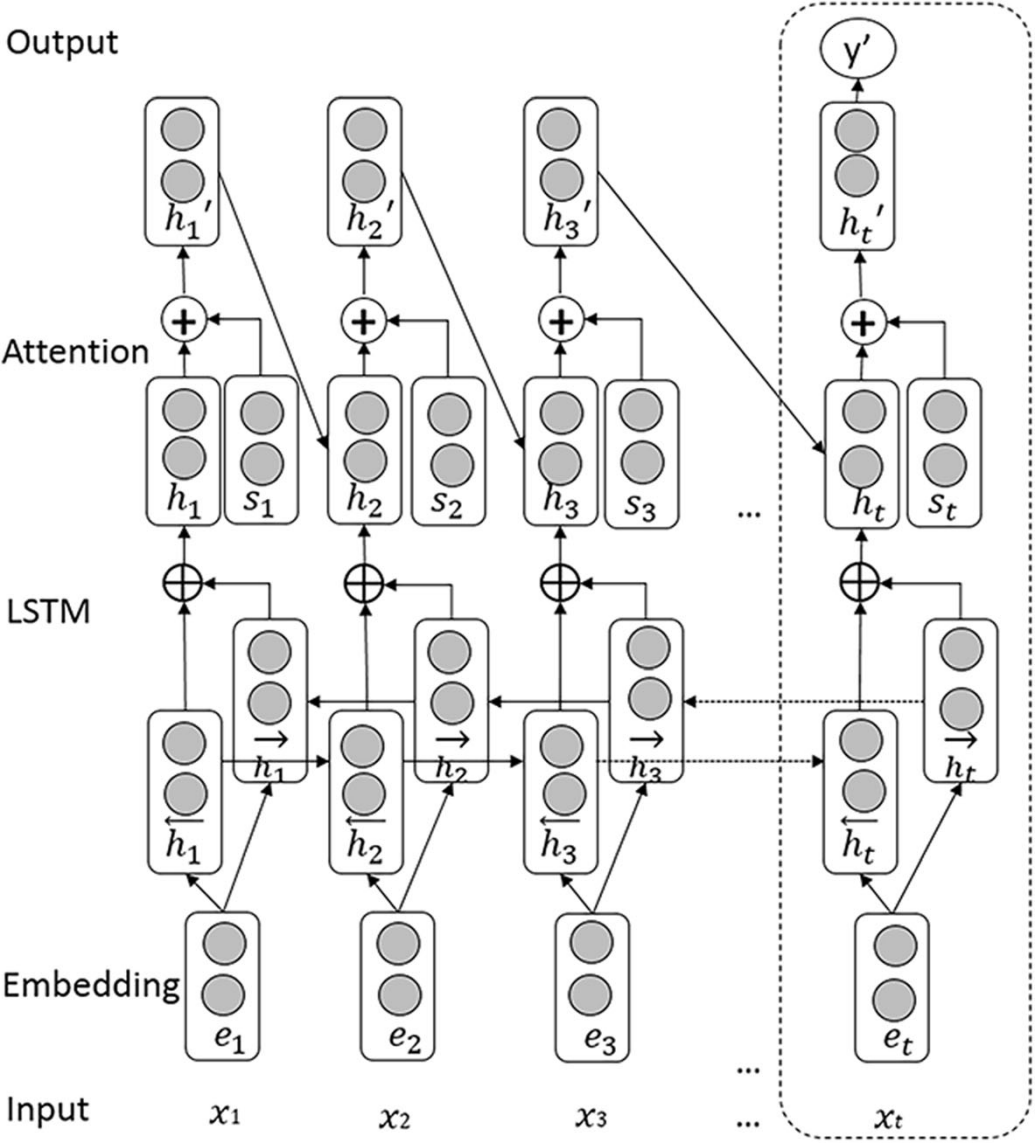
BiLSTM model for the entity and relation extraction. The flow chart of the BiLSTM network to predict the type of the entity and relation

Before training the BiLSTM model to predict the type of each entity, we need to predict the token’s position tag of each word. Each entity is treated as a unit input. We calculate the entity vectors using by adding the entity word vector and the type vector with the annotation vector and then use the average value of all the word embeddings of an entity in the next process. The part of the network in the dotted box in Fig. 6 shows the specific flow chart of the BiLSTM network to predict the type of the entity. In this process, the prediction of the entity’s class is predicted from the given input entity vector.

### Semantic relation extraction

We utilize the proposed approach to extract biomedical semantic relations, which is the primary purpose of this paper. As shown in Fig. 6, the system contains five components. (1) Input layer: the input can be formed in two ways, one is the whole sentence containing the entity pairs, and the other way is that we select, we select the sentence between the entity pairs as the input sentence. The purpose is to prevent the LSTM network from forgetting the information of the critical entities in the sentence due to too long sentences. However, some entity pairs are very close to each other, so they cause too short sentences. Therefore, we expand the window size by two words to cover the terms before and after the entity pair. (2) Embedding layer: map each word into a vector. (3) BiLSTM layer: two hidden states at the time step, which can be viewed as a combination of the past and future information. (4) Attention mechanism: leading in the KB information from BioModels and filtering the information with the weight vector to reflect how external information is relevant to the current state *ht*. Merging word-level features from each time step into a sentence-level feature vector is also done in this step. (5) Output layer: the sentence-level feature vector is used to classify the relationship type.

## Data Availability

The datasets can be obtained from the BioNLP and BioCreative website. The dataset employed is available at http://2016.bionlp-st.org/ and http://www.biocreative.org/tasks/biocreative-vi/track-4/

## Abbreviations

PPI: Protein-protein interactions
KB: Knowledge base
BiLSTM: Bidirectional long short-term memory
LSTM: Long short-term memory
SVM: Supper vector machine
AttBLSTM: Attention-based bidirectional long short-term memory networks
NER: Named entity recognition
POS: Part-of-speech

## Footnotes

1 https://thebiogrid.org/

2 https://www.ebi.ac.uk/intact/

3 http://www.uniProt.org/

4 https://www.ebi.ac.uk/biomodels/

5 https://dip.doe-mbi.ucla.edu/dip/Main.cgi

6 http://2016.bionlp-st.org/ tasks/seedev.

7 https://pypi.org/project/bioservices/

8 https://docs.python.org/2/library/urllib.html

9 https://www.crummy.com/software/BeautifulSoup/bs4/doc/index.zh.html
